# Selective migration and mortality by economic status in Lolland-Falster, Denmark, 1992–2018

**DOI:** 10.1038/s41598-022-24635-2

**Published:** 2022-11-19

**Authors:** Therese Lucia Friis Holmager, Søren Nymand Lophaven, Laust Hvas Mortensen, Elsebeth Lynge

**Affiliations:** 1 Centre for Epidemiological Research, Nykøbing Falster Hospital, Ejegodvej 63, 4800 Nykøbing Falster, Denmark; 2 Omicron ApS, Sankt Jørgens vej 15, 4000 Roskilde, Denmark; 3 Statistics Denmark, Sejrøgade 11, 2100 Copenhagen, Denmark

**Keywords:** Epidemiology, Public health

## Abstract

During the past 30 years, a mortality gap developed between Lolland-Falster (the rural-provincial southeastern part) and the rest of Denmark. A main driver was selective in-migration of Danes with a high risk of death, especially of working-ages. In the present study, we determined the role of economic status in this selective in-migration. We used individual-level data from the Central Population Register and data on income source; self- or publicly supported. The study population included people aged 30–64 and living in Denmark at any time between 1992 and 2018. Mortality rate ratios (MRR) were calculated using Poisson regression for three time-periods: 1992–1999, 2000–2009 and 2010–2018. Two in five in-migrants to Lolland-Falster were people on public support. In 2010–2018, they had an MRR of 8.71 (95% confidence interval (CI): 8.05–9.42) compared with self-supported people, and an MRR of 1.49 (95% CI: 1.38–1.61) compared with publicly supported people elsewhere in Denmark. In-migration of working-aged people on public support was a main contributor to the excess mortality in Lolland-Falster as compared with the rest of Denmark. To understand urban–rural differences in health, population movements and national income patterns are important to take into account.

## Introduction

Trends of healthy people leaving rural areas in favour of urban areas have been found to increase geographical inequalities in health, as the vulnerable and elderly people are left behind^1–4^. In Europe, certain rural areas suffering from depopulation may enter a ‘vicius circle of decline’ where people migrate in search of better jobs, public health services decline, and private health service practioners move to more profitable areas^5^.

In Denmark, Lolland-Falster is a rural-provincial area in the south eastern part of the country characterised by depopulation^6^, low life expectancy^7^, a high proportion of elderly people^6^, several negative health indicators such as high prevalence of smoking and obesity, and low prevalence of physical activity, and a high prevalence of chronic diseases e.g. diabetes, hypertension and cancer^8^.

However, in contrast to the observations from other countries, the currently high mortality in Lolland-Falster has been driven mainly by vulnerable people of working-age moving into Lolland-Falster from other parts of Denmark^9,10^. In-migrants to Lolland-Falster of working-ages have a mortality rate ratio (MRR) of 2.35 (95% confidence interval (CI): 2.19–2.52) compared with the mortality in the rest of Denmark^10^.

On this basis, we determined the role of economic status behind this selective migration pattern from the urban parts of Denmark to the rural-provincial area of Lolland-Falster.

## Methods

The study population included all people aged 30–64 years and living in Denmark at any time between 1992 and 2018. Data for the study population were extracted from the Central Population Register (CPR), with individual-level data on all persons ever living in Denmark in the study period including data on sex, date of birth, death, address history, emigration, and immigration^11^. A given person contributed person-years at risk during the time-period he/she had been present in Denmark in the relevant age-group 30–64 years. We studied three time-periods: 1992–1999, 2000–2009; and 2010–2018.

CPR data were merged with data from Statistics Denmark´s socioeconomic classification (SOCIO13) including data on annual main income source for 1991–2017^12^. Data was accessed through Statistics Denmark’s research server. Data are available from Statistics Denmark upon reasonable request.

### Residency group

Lolland-Falster includes Lolland and Guldborgsund municipalities. We divided the Danish population into three residency groups: long-term residents of Lolland-Falster, the in-migrants to Lolland-Falster, and the rest of the Danish population for the three time-periods (1992–1999, 2000–2009, and 2010–2018). Construction of the residency groups has been reported in details previously^10^. In short, a person’s residency group was based on his/her address history during the previous 10 years. Thus, to be a long-term resident in a given time-period, a person had to have lived continuously in Lolland-Falster 10 years prior to the time-period and he/she remained in the long-term residency group up until death, emigration, or end of follow-up. In-migrants had moved to Lolland-Falster during the 10 years preceding the time-period or during the time-period. A person could contribute person-years to different residency groups during different time-periods, and a long-term resident would become an in-migrant if he/she moved outside Lolland-Falster and back again during a time-period. The long-term residents and the in-migrants together constituted the total Lolland-Falster population.

### Economic status

In a previous study on economic status in Danish municipalities by the Danish Center for Social Science Research, unemployed persons and other people on public support were merged into one group^13^. Likewise, in the present study we used the annual SOCIO13-groups with 20 mutually exclusive categories to create a dichotomous variable indicating whether a person during the majority of the previous year was economically self-supported or publicly supported. The self-supported group included employees, self-employed persons and their assisting spouses. The publicly supported group included people on public transfer payment; non-old age pensioners, old age pensioners, and people on unemployment benefit, early retirement scheme, sickness benefits, cash benefits or adult education support (see Appendix Table 1)^12^. Non-old age pensioners included persons receiving pension due to disability or chronic disease. Cash benefit is the residual group of the unemployed people not qualified to be in the other groups, including individuals waiting to be accepted into one of the other groups. If a person had missing data for SOCIO13 one year; they would not contribute to the analysis with person-years or deaths in the following year.

### Analysis

The study used Danish administrative register-data. All registers include date of birth, all changes of addresses with dates, and data on socioeconomic status are registered once a year. The study population was dynamic in the sense that a given person contributed person-years to a given group from the point in time where he/she fulfilled the criteria for belonging to the group until he/she did not any longer fulfill these criteria. First, a given person contributed person-years as long as he/she was 30–64 years old. Second, a given person belonged to the population in the rest of Denmark as long as he/she was registered with an address in Denmark outside Lolland-Falster. Lolland-Falster residents were divided into long-term residents and in-migrants based on their address history. Third, a given person belonged to the last socioeconomic group in which he/she appeared in the register.

The number of persons belonging to a given group can be calculated for a given date, e.g. 1 January 1992. However, over time a given person could contribute to more than one group. The sum of numbers of persons aged 30–64 years over time, e.g. 1992 to 1999, contributing to the different groups was therefore larger than the counts of persons on a given date. The population flow by address-group during each time-period is illustrated in Appendix Fig. 1. Due to movements between groups over time, the study population is reflected better in person-years than in the number of persons.

For analysis of mortality by residency and economic status, we used Poisson regression to calculate mortality rate ratio (MRR) with 95% CI. We used the method described by Rostgaard to transform the longitudinal dataset into event-time tables and conduct the Poisson regression^14^. The analysis was conducted separately for men and women and for the total population. The model was adjusted for 5-year age-group and for sex for the total population. For each time-period, self-supported people in the rest of Denmark were used as the reference group; or publicly supported people in Lolland-Falster were compared with that of publicly supported people in the rest of Denmark.

Two sensitivity analyses were made. First, residency was categorised by residency 20 years instead of 10 years before each time-period. Second, as a self-supported person who becomes sick and dies may receive public support shortly before death, the death rate for publicly supported persons may be inflated. Thus, an additional analysis was made categorising economic status by the annual status three years before. This analysis could not be made for 1992–1999, as SOCIO13 data were available only from 1991 onwards.

All analysis were made in SAS 9.4 and graphics were made in R 4.0.3 at a research server in Statistics Denmark.

### Ethics

The present study was a register-based study using anonymised individual level data. According to Danish legislations, register-based studies not including genetic data and with no contact to patients, relatives, and/or treating physician, registration by the responsible institution serves at ethical clearance (*Databeskyttelsesforordningen* and *Databeskyttelsesloven*)^15,16^. The study registration number in Region Zealand is REG-108-2018.

## Results

**Figure 1:**
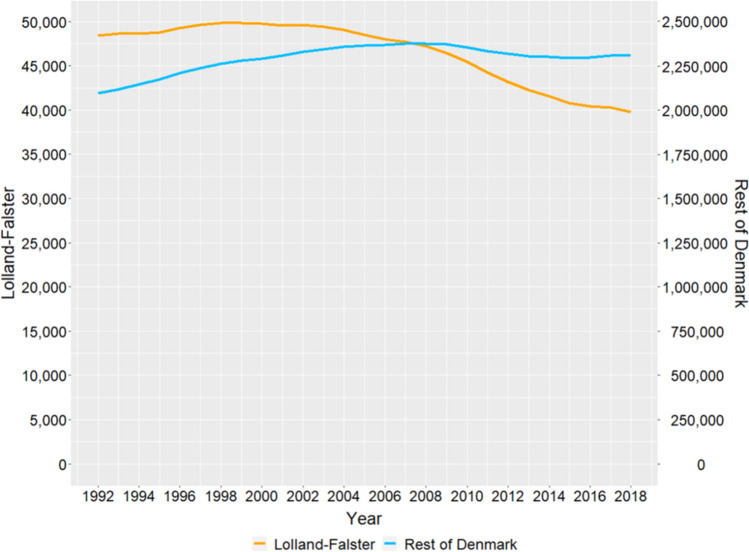
Mid-year population number for people aged 30–64 years living in Lolland-Falster and in the rest of Denmark by year, 1992–2018.

In the rest of Denmark, the population aged 30–64 increased between 1992 and 2008 from 2,090,000 to 2,380,000; decreased to 2,300,000 in 2013, and was relatively stable until 2018 (Fig. 1). In Lolland-Falster, the population aged 30–64 increased slightly from 48,400 in 1992 to 49,800 in 1998, whereafter it decreased by 20% to 39,800 in 2018.

The study population included 66,226,171 person-years and 270,466 deaths (Table 1). In the rest of Denmark, the self-supported group constituted 74% of the person-years in 1992–1999, and 78% in 2000–2009 as well as 2010–2018. In Lolland-Falster, the self-supported group constituted 67% of the person-years in 1992–1999, 70% in 2000–2009, and 68% in 2010–2018. In Lolland-Falster in 2010–2018, 72% of long-term residents and 58% of in-migrants in Lolland-Falster were self-supported.

**Table 1:** Number of person-years and deaths for people aged 30–64 years by residency group, economic status, and time-period.

	Self-supported		Public support		Total	
	Person-years, n (%)	Deaths	Person-years, n (%)	Deaths	Person-years, n	Deaths
**2010–2018**						
Rest of Denmark	16,773,066 (78%)	25,041	4,637,636 (22%)	47,057	21,410,702	72,098
Lolland-Falster	289,617 (68%)	680	134,652 (32%)	1839	424,269	2519
In-migrants	69,107 (58%)	126	50,733 (42%)	642	119,840	768
Long-term residents	220,510 (72%)	554	83,919 (28%)	1197	304,429	1751
Total	17,062,683 (78%)	25,721	4,772,289 (22%)	48,896	21,834,972	74,617
**2000–2009**						
Rest of Denmark	19,044,254 (78%)	36,970	5,514,448 (22%)	63,845	24,558,702	100,815
Lolland-Falster	380,723 (70%)	1006	166,389 (30%)	2476	547,112	3482
In-migrants	93,218 (59%)	228	63,833 (41%)	952	157,051	1180
Long-term residents	287,505 (74%)	778	102,556 (26%)	1524	390,061	2302
Total	19,424,977 (77%)	37,976	5,680,837 (23%)	66,321	25,105,814	104,297
**1992–1999**						
Rest of Denmark	13,660,948 (74%)	31,545	4,750,633 (26%)	54,857	18,411,581	86,402
Lolland-Falster	291,713 (67%)	768	145,189 (33%)	1807	436,902	2575
In-migrants	58,942 (54%)	136	49,382 (46%)	611	108,324	747
Long-term residents	232,771 (79%)	632	95,807 (29%)	1196	328,578	1828
Total	14,244,374 (74%)	33,081	5,041,011 (26%)	58,471	19,285,385	91,552
Total	50,732,034 (77%)	96,778	15,494,137 (23%)	173,688	66,226,171	270,466

Over time, self-supported, long-term residents constituted 52–54% of the person-years in the total Lolland-Falster population, while self-supported in-migrants constituted 13–17%. The publicly supported, long-term residents constituted 19–22% of the total Lolland-Falster population, while the publicly supported in-migrants constituted the remaining 11–12%. The publicly supported group consisted mainly of persons on non-old age pension or cash benefit. In 2010–2018, 47% of people on public support in Lolland-Falster received non-old age pension; 51% among long-term residents and 39% among in-migrants; compared with 37% in the rest of Denmark (Appendix Table 1). The percentages for cash benefit were 21% for Lolland-Falster; 15% for long-term residents; 31% for in-migrants; and 20% for the rest of Denmark.

### Mortality rate ratio for residency groups by economic status

**Figure 2:**
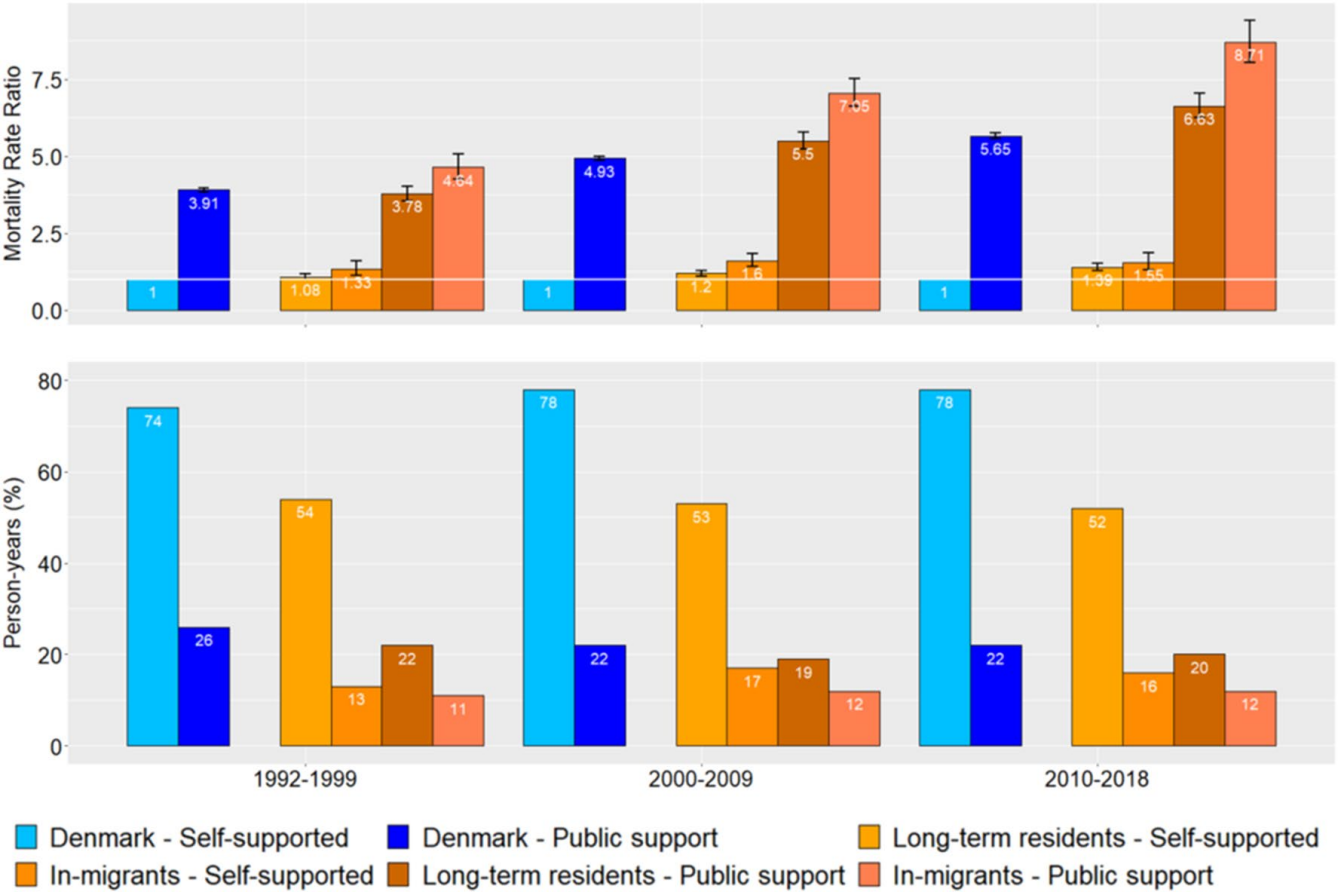
Mortality rate ratio and percentage of person-years for the rest of Denmark and the total population aged 30–64 years in Lolland-Falster by residency group, economic status, and time-period. Adjusted for 5-year age-groups and sex. Reference population = Self-supported in rest of Denmark.

Compared with self-supported people in the rest of Denmark, self-supported people in Lolland-Falster had an elevated MRR increasing by time from 1.12 (95% CI: 1.04–1.21) in 1992–1999 to 1.42 (95% CI: 1.31–1.53) in 2010–2018 (Table 2 and Fig. 2). Compared with publicly supported people in the rest of Denmark, publicly supported people in Lolland-Falster had an elevated MRR increasing from 1.02 (95% CI: 0.98–1.07) in 1992–1999 to 1.28 (95% CI: 1.22–1.34) in 2010–2018 (Appendix Table 2).

**Table 2:** Person-years, number of deaths and mortality rate ratio for people aged 30–64 years in Lolland-Falster by residency group, economic status, sex, and time-period.

	Self-supported			Public support			Total		
	Person-years	Deaths	MRR	Person-years	Deaths	MRR	Person-years	Deaths	MRR
**2010–2018**									
Men									
Rest Denmark	8,646,953	15,626	1	2,069,044	28,895	6.22 (6.09–6.34)	10,715,996	44,521	2.10 (2.07–2.13)
Lolland-Falster	152,071	431	1.43 (1.30–1.58)	64,748	1168	7.90 (7.44–8.39)	216,818	1599	3.28 (3.14–3.41)
In-migrants	38,164	91	1.69 (1.38–2.08)	25,747	440	9.76 (8.88–10.74)	63,911	531	4.98 (4.64–5.35)
Long-term residents	113,907	340	1.38 (1.24–1.53)	39,001	728	7.08 (6.58–7.63)	152,908	1068	2.85 (2.71–2.99)
Women									
Denmark	8,126,114	9415	1	2,568,593	18,162	4.80 (4.68–4.93)	10,694,706	27,577	2.01 (1.97–2.06)
Lolland-Falster	137,546	249	1.40 (1.23–1.58)	69,904	671	6.18 (5.72–6.69)	207,450	920	3.03 (2.83–3.24)
In-migrants	30,943	35	1.30 (0.94–1.82)	24,987	202	7.01 (6.10–8.06)	55,929	237	4.19 (3.69–4.77)
Long-term residents	106,603	214	1.41 (1.23–1.62)	44,918	469	5.89 (5.36–6.46)	151,521	683	2.77 (2.56–2.99)
Total									
Denmark	16,773,066	25,041	1	4,637,636	47,057	5.65 (5.56–5.74)	21,410,703	72,098	2.15 (2.11–2.19)
Lolland-Falster	289,617	680	1.42 (1.31–1.53)	134,652	1839	7.24 (6.90–7.59)	424,269	2519	3.43 (3.26–3.61)
In-migrants	69,107	126	1.55 (1.30–1.85)	50,733	642	8.71 (8.05–9.42)	119,840	768	5.44 (4.99–5.94)
Long-term residents	220,510	554	1.39 (1.28–1.51)	83,919	1197	6.63 (6.26–7.03)	304,429	1751	2.89 (2.72–3.08)
**2000–2009**									
Men									
Denmark	10,053,788	23,997	1	2,328,834	38,053	5.36 (5.27–5.46)	12,382,622	62,050	1.92 (1.89–1.95)
Lolland-Falster	204,578	679	1.31 (1.21–1.41)	75,642	1567	6.59 (6.26–6.93)	280,220	2246	2.81 (2.69–2.94)
In-migrants	52,121	163	1.68 (1.44–1.96)	31,421	638	7.60 (7.02–8.22)	83,542	801	4.30 (4.00–4.61)
Long-term residents	152,458	516	1.22 (1.12–1.34)	44,220	929	6.04 (5.65–6.45)	196,678	1445	2.36 (2.24–2.49)
Women									
Denmark	8,990,465	12,973	1	3,185,614	25,792	4.23 (4.14–4.33)	12,176,080	38,765	1.89 (1.86–1.93)
Lolland-Falster	176,145	327	1.20 (1.07–1.33)	90,747	909	5.06 (4.73–5.42)	266,892	1236	2.51 (2.37–2.66)
In-migrants	41,097	65	1.44 (1.13–1.83)	32,412	314	6.09 (5.45–6.81)	73,509	379	3.66 (3.30–4.05)
Long-term residents	135,048	262	1.15 (1.02–1.30)	58,335	595	4.65 (4.28–5.05)	193,383	857	2.20 (2.05–2.36)
Total									
Denmark	19,044,254	36,970	1	5,514,448	63,845	4.93 (4.86–4.99)	24,558,702	100,815	1.91 (1.89–1.93)
Lolland-Falster	380,723	1006	1.27 (1.19–1.35)	166,389	2476	6.01 (5.77–6.26)	547,112	3482	2.70 (2.61–2.79)
In-migrants	93,218	228	1.60 (1.40–1.82)	63,833	952	7.05 (6.61–7.52)	157,051	1180	4.07 (3.84–4.31)
Long-term residents	287,505	778	1.20 (1.11–1.29)	102,556	1524	5.50 (5.22–5.79)	390,061	2302	1.20 (1.16–1.26)
**1992–1999**									
Men									
Denmark	7,351,405	20,888	1	1,929,695	31,394	4.28 (4.19–4.36)	9,281,100	52,282	1.77 (1.74–1.80)
Lolland-Falster	159,552	509	1.12 (1.02–1.23)	62,537	1080	4.41 (4.13–4.71)	222,089	1589	2.09 (1.99–2.20)
In-migrants	33,619	92	1.32 (1.06–1.63)	24,476	403	4.97 (4.47–5.53)	58,095	495	3.03 (2.77–3.32)
Long-term residents	125,932	417	1.09 (0.98–1.20)	38,061	677	4.14 (3.82–4.49)	163,994	1094	1.83 (1.72–1.95)
Women									
Denmark	6,309,543	10,657	1	2,820,938	23,463	3.32 (3.24–3.41)	9,130,481	34,120	1.80 (1.76–1.84)
Lolland-Falster	132,161	259	1.12 (0.98–1.27)	82,652	727	3.40 (3.14–3.68)	214,813	986	2.02 (1.89–2.16)
In-migrants	25,322	44	1.40 (1.02–1.93)	24,906	208	4.02 (3.47–4.67)	50,229	252	2.82 (2.49–3.19)
Long-term residents	106,839	215	1.07 (0.93–1.24)	57,745	519	3.21 (2.92–3.52)	164,584	734	1.84 (1.71–1.99)
Total									
Denmark	13,660,948	31,545	1	4,750,633	54,857	3.91 (3.84–3.97)	18,411,581	86,402	1.78 (1.76–1.80)
Lolland-Falster	291,713	768	1.12 (1.04–1.21)	145,189	1807	4.02 (3.82–4.23)	436,902	2575	2.06 (1.98–2.15)
In-migrants	58,942	136	1.33 (1.12–1.59)	49,382	611	4.64 (4.25–5.06)	108,324	747	2.96 (2.75–3.18)
Long-term residents	232,771	632	1.08 (0.99–1.17)	95,807	1196	3.78 (3.55–4.02)	328,578	1828	1.84 (1.75–1.93)

Mortality rate ratios were adjusted for 5-year age-groups and sex. Self-supported in rest of Denmark is the reference population.

For the self-supported group in Lolland-Falster, the excess mortality as compared with that of self-supported people in the rest of Denmark emerged over time for long-term residents and was present for in-migrants in all time-periods (Table 2 and Fig. 2). The MRRs for self-supported, long-term residents increased from 1.08 (95% CI: 0.99–1.17) in 1992–1999 to 1.39 (95% CI: 1.28–1.51) in 2010–2018. For self-supported in-migrants, the MRR increased from 1.33 (95% CI: 1.12–1.59) in 1992–1999 to 1.55 (95% CI: 1.30–1.85) in 2010–2018.

For the publicly supported group in Lolland-Falster, the excess mortality as compared with self-supported people in the rest of Denmark increased with every time-period for the long-term residents as well as for the in-migrants. The MRR for the publicly supported long-term residents reached 6.63 (95% CI: 6.26–7.03) in 2010–2018, and the MRR for publicly supported in-migrants reached 8.71 (95% CI: 8.05–9.42) in 2010–2018. However, compared with self-supported people, the excess mortality for the publicly supported group increased also over time in the rest of Denmark from 3.91 (95% CI: 3.84–3.97) in 1992–1999 to 5.65 (95% CI: 5.56–5.74) in 2010–2018.

The MRR trends were similar for men and women. It should be noted though that in 2010–2018 the MRR for male in-migrants on public support reached 9.76 (95% CI: 8.88–10.74).

### Sensitivity analysis

Sensitivity analysis using 20 years of address history prior to the time-period to categorise the residency groups, showed similar results as the main analysis using 10 years of address history (Appendix Table 3).

When categorising annual economic group three years back in time instead of one year, the excess mortality for publicly supported people compared with self-supported people decreased slightly (Appendix Table 4). Compared with self-supported people in the rest of Denmark, the MRR for publicly supported people in the rest of Denmark 2010-2018 was 5.65 (95% CI: 5.56–5.74) when categorising one year back in time and 4.60 (95% CI: 4.53–4.67) when categorised three years back. For the total of Lolland-Falster, the change was from an MRR of 7.24 (95% CI: 6.90–7.59) to 6.08 (95% CI: 5.78–6.40).

## Discussion

### Main results

Mortality in the rural-provincial area of Lolland-Falster is high compared with the level in the rest of Denmark. This is a relatively new phenomenon where a gap in mortality between Lolland-Falster and the rest of Denmark emerged gradually from around 1990^9^. We have shown previously that this excess mortality derived mainly from in-migrants from other parts of Denmark, and especially from in-migrants of working-age^10^. In the present analysis, we demonstrated that this mortality gap was attributable mainly to in-migration to Lolland-Falster of persons economically on public support. In the Danish welfare system, poor health is in some instances a precondition for eligibility for public support in working-age^17^. In 2010–2018, two out of five in-migrants of working-age to Lolland-Falster were on public support, and these in-migrants had a ninefold mortality compared with that of self-supported persons in the rest of Denmark. Selective migration of publicly supported people of working-age was thus the most important factor behind the presently high mortality at Lolland-Falster.

### Previous studies

The impact of selective migration on area-based differences in mortality has been investigated in a number of studies from the UK. Most of these studies have taken advantage of the Office for National Statistics Longitudinal Study (ONS LS)^18^. In these studies, areas have been classified in two ways; by an area-based deprivation index and by a rural–urban gradient. When comparing studies from the UK with those from other countries, it is important to note that there is a positive correlation between severity of deprivation and urbanity in the UK, while in many other countries, the rural areas are the most deprived. In the following we therefore focus on the UK studies on rural–urban differences in mortality.

It was a consistent pattern across the UK studies that rural areas had lower mortality than urban areas, and this mortality gap increased over time^19^. Over the 20-year period from 1981 to 2001, selective migration of better off people from urban to rural areas contributed to this increasing gap in England and Wales^19^, but in studies covering shorter periods; 1991–2001 in Scotland^20^ and 2006–2011 in Wales^21^, selective migration had no impact on the regional mortality differences. As noted by Brimblecombe et al.^22^, the measured impact of migration on geographical differences in mortality depends also on the composition of the areas; the measured impact will be greater when migration is studied between small homogeneous districts than between larger heterogenous regions.

In Sweden, the mortality of people moving from Northern to Southern Sweden resembled that of people staying in other parts of Sweden^23^. However, the movers had higher education, thus, when adjusting for education, the movers had an excess mortality. Men returning to Northern Sweden after having lived in Southern Sweden had higher mortality than men staying in other parts of Sweden. Adjustment for education enhanced the difference. The pattern illustrated the salmon effect where migrants move home in anticipation of death; here the pattern indicated to be most common among migrants with higher education.

Another Swedish study investigated the potential impact of selective migration on the low mortality in Halland, Sweden for the period 1980–1990^24^. Within Halland, men born there had lower mortality than men born elsewhere despite indication of a higher socioeconomic background of these in-migrants^25^. Outside Halland, men born in Halland still had lower mortality than other men. These differences were statistically non-significant for women^24^. Thus, selective migration did not explain the low mortality in Halland.

Mortality in 1971–2004 for 40–59 old persons in Finland was analysed by birthplace^26^. Among men living in Southern Finland, those born in Northern and Eastern Finland had an excess mortality. When adjusted for education the excess risk prevailed for men born in Northern Finland but not for men born in Eastern Finland. Men who migrated from Northern Finland also had higher mortality than men who stayed in the North. Differences for women were not statistically significant. The study indicated that men who migrated out of Northern Finland constituted a high-risk group unrelated to their level of education.

The difference between the UK and Denmark was noteworthy. In the UK, the rural areas have lower mortality than urban areas, and selective migration of better off people from urban to rural areas contributed to a widening of this gap over time. In Denmark, the rural area of Lolland-Falster has higher mortality than the rest of Denmark, and selective in-migration of people on public support has contributed to a widening of this gap. The Swedish and Finnish studies focused on people moving from the rural North to the urban South of the respective countries. In Finland, people in the North had higher mortality than people in the South and Northern migrants to the South constituted a high-risk group both compared with those who stayed in the North and those who lived in the South. In Sweden, migrants from the North had a mortality similar to people staying in the South, while movers from the North returning to the North had a higher risk of death. In Denmark, the mortality gap between Lolland-Falster and the rest of Denmark started around 1990^9^. The mortality gap developed alongside with a population decrease. The decrease has mainly been attributed to young people moving away^10^, but these generations are still too young to have affected regional differences in mortality.

### Strengths and limitations

A strength in the present study was the use of nationwide, high-quality, Danish register data. As these register-data are used for official statsistics, they are already validated by Statistics Denmark, before released for research purpose. On a subject level we divided the follow-up time into groups given by age, calendar period, sex, residency group, and economic status and counted the number of deaths for each combination of these variables. The natural model for analysing count data is Poisson regression. Differences in follow-up time were accounted for by including an offset in the model^27^.

Mortality was higher among people on public support than among self-supported people, but the mortality rates of people on public support may be inflated by deaths in previously self-supported people being on e.g. sickness benefit towards the end of life. We therefore added an analysis based on economic status three years back (see Appendix Table 4). This decreased the excess mortality of publicly supported people, though the relative difference between Lolland-Falster and the rest of Denmark, between in-migrants and long-term residents, and across time-periods persisted.

### Interpretation

The above comparison with UK and Nordic studies illustrated that local circumstances have to be taken into account in the interpretation of regional differences in mortality. In the rural area of Lolland-Falster, the mortality is higher than in the rest of Denmark, and the present study showed that a major part of this excess mortality is attributable to in-migrants on public support.

The increased mortality among people on public support may be due to selection with illness causing inability to work and/or a negative impact on health of a life outside the labour market^28^. In our study population, the most common type of public support was non-old age pension for which permanently impaired health is the main eligibility criteria^17^. Among publicly supported people, those on unemployment benefit constituted a smaller group, but unemployment in itself may impair health^29^. The excess mortality of the publicly supported group in our study increased over time. This trend may have been enhanced by political initiatives to keep people in the workforce and affecting selection as more stringent criteria were used for granting of non-old age pension. It may also explain the increasing proportion of people on cash benefit, which is granted to people in principle considered suitable for work. Cash benefit is the economically lowest type of public support. Thus, the publicly supported people in Denmark may have become relatively poorer over time compared with the self-supported people.

Economic Council of the Labour Movement has previously reported that Lolland-Falster was among the areas in Denmark with the highest net growth in in-migration of people on public support^30^, and in 2020, in-migrants to Lolland-Falster had a lower disposable income than in-migrants to other municipalities^31^. A reason for people with a relatively low disposable income moving to Lolland-Falster was primarily an increasing gap in housing prices between rural areas like Lolland-Falster and urban/suburban areas close to the capital of Copenhagen^32,33^. In Denmark, people on public support can move freely between areas of the country. Thus, it was possible for publicly supported people to move to an area with lower expenses. Other changes in living circumstances like break-up with partners, conflicts with neighbors or other difficulties in life that we could not include in the present study, may also have played a role. The in-migrant population was not stable but relatively dynamic with persons moving in, followed by moving out, and then in again and out again, as illustrated in Appendix Fig. 1. The disposable income decreased from 2002 to 2011 for the poorest decile of the Danish population, despite an increase in the average income^34^, and using the Gini coefficient the OECD concluded that Denmark among other countries experienced increasing income inequality between 1980 and 2013^35^. Low socioeconomic status is in itself a risk factor for premature death^36^, and social inequality in mortality in Denmark has increased^37,38^.

We observed previously a selective in-migration to Lolland-Falster of people with a high risk of death^10^. The present study was the first to investigate the role of economic status in selective migration and regional mortality differences in Denmark. Given the high inflow of people at a high risk of death, our study illustrates the role of circulation of biological capital^39^ in the generation of regional differences in mortality in a Nordic welfare state. However, the excess mortality on Lolland-Falster could not be explained entirely by the large share of people on public support. Compared with the rest of Denmark, we found an excess mortality in Lolland-Falster within each of the economic status and residency groups, though stronger for in-migrants than for long-term residents. An explanation could be that in-migrants generally have a weaker social support system than long-term residents, placing them at increased risk of death when health fails them. Another explanation could be a generally negative health selection of people moving to Lolland-Falster independently of economic status. It should be noted though that we used a crude categorisation of economic status; within the group of self-supported people there is inequalities in mortality across occupations^40,41^; and within the group of publicly supported people, mortality is expected to differ between those on temporary unemployment benefit and those on permanent non-old age pension.

Lolland-Falster had a relatively high annual population turn-over of 7%^42^. The number of people moving in and out of Lolland-Falster was almost similar over the years, though, a higher number of people moving in were between ages 30 and 64 years^42^. We found previously that long-term residents moving away from Lolland-Falster were younger and had lower mortality than in-migrants moving out^10^. However, as out-migrants are relatively young, the absolute number of deaths in this group is small, and mortality estimates unstable. We therefore did not include sub-analysis by socioeconomic groups for out-migrants.

Denmark is a country where fifty years ago a high mortality was found in the inner-city working-class areas, especially in the capital of Copenhagen^43^. Otherwise throughout the rest of Denmark, health conditions and mortality were fairly similar. Over time, this pattern has been reversed. At present the highest mortality in Denmark is found in the rural area of Lolland-Falster. Our study demonstrated that health-related internal migration was a key component in this reversal. High prices of housing in the capital and suburban areas pushed people on public support, and therefore on low income, to move to areas like Lolland-Falster where prices were lower. This led to an aggregation in Lolland-Falster of unstable people at a high risk of death. The high influx of vulnerable people to Lolland-Falster places a burden on the health care and social support systems in the area. The in-migrants die from the same causes as people in the rest of Denmark, just at a younger age^44^. Thus, health prevention and treatment in Lolland-Falster cannot be restricted to specific causes; nor to this geographical area as many of the in-migrants’ health complications were established elsewhere.

## Conclusion

Economic status was a key factor behind the increasing mortality gap between Lolland-Falster and the rest of Denmark. Two out of five in-migrants to Loland-Falster were on public support, and they had a nine-fold death rate as compared with self-supported people in the rest of Denmark, and a 50% higher mortality than publicly supported people elsewhere in Denmark. Also, self-supported in-migrants to Lolland-Falster was a somewhat selected group with a 50% higher mortality than self-supported people in the rest of Denmark. Over time, this inflow of vulnerable people affected the mortality pattern of long-term residents. While the self-supported long-term residents in 1992–1999 had the same mortality as self-supported people in the rest of Denmark, they had by 2010–2018 a 40% excess mortality. In Denmark, poor health is the main eligibility criteria for public support in working-ages. Thirty years of inflow of this healthwise vulnerable group of citizens affected the mortality generating a gap between Lolland-Falster and the rest of Denmark.

## Supplementary Information

Supplementary Information.

## Data Availability

Data was extracted from the Danish Health Data Authority and Statistics Denmark. The data that support the findings of this study are available from Statistics Denmark but restrictions apply to the availability of these data, which were used under license for the current study, and so are not publicly available. Data used for this study can be accessed from Statistics Denmark’s Research Service upon reasonable request (e-mail: forskningsservice@dst.dk).
