# Supplementary material for: Selective migration and mortality by economic status in Lolland-Falster, Denmark, 1992–2018
Source: Sci Rep. 2022 Nov 19;12:19970. doi: 10.1038/s41598-022-24635-2 (PMC9675768; doi:10.1038/s41598-022-24635-2)
Supplement: Supplementary file 1 — Supplementary Information. [file 41598_2022_24635_MOESM1_ESM.pdf]

Supplementary Material: Selective migration and mortality by economic status in Lolland-Falster, Denmark, 1992-2018

Therese Lucia Friis Holmager [a]*, Søren Nymand Lophaven [b],
Laust Hvas Mortensen [c], Elsebeth Lynge [a]

a) Centre for Epidemiological Research, Nykøbing Falster Hospital, Ejegodvej 63, DK-4800 Nykøbing Falster, Denmark
b) Omicron ApS, Sankt Jørgens vej 15, DK-4000 Roskilde, Denmark
c) Statistics Denmark, Sejrøgade 11, DK-2100 Copenhagen, Denmark

Appendix Table 1: SOCIO13 groups for economic status. Person-years, % of total for residency groups

		Rest of Denmark	Lolland-Falster	In-migrants	Long-term residents
	**2010-2018**				
Self-supported	Self-employed (≥10 employees)	0.09%	0.14%	0.08%	0.16%
	Self-employed (5-9 employees)	0.22%	0.27%	0.13%	0.31%
	Self-employed (1-4 employees)	1.33%	1.77%	0.93%	2.03%
	Self-employed (0 employees)	5.00%	5.30%	4.94%	5.41%
	Assisting spouses	0.19%	0.21%	0.09%	0.25%
	Employee with management work	4.59%	3.15%	2.80%	3.26%
	Employee in work that requires skills at the highest level	17.63%	9.95%	14.40%	8.55%
	Employee in work that requires skills at medium level	21.48%	19.09%	18.06%	19.41%
	Employee in work that requires skills at the basic level	33.35%	41.03%	38.33%	41.87%
	Other employees	7.13%	9.54%	9.00%	9.71%
	Employee, position statement not stated	8.99%	9.56%	11.23%	9.03%
Public supported	Unemployed at least half of the year	9.62%	6.57%	7.67%	5.90%
	Sickness benefits, leave, etc.	6.62%	4.73%	5.53%	4.25%
	Students	5.20%	2.66%	4.41%	1.59%
	Non-old age pensioners	36.88%	46.42%	38.96%	50.94%
	Old age pensioners	0.00%	0.00%	0.00%	0.00%
	Early retirement scheme	12.15%	11.76%	3.63%	16.67%
	Recipients of cash benefits	19.96%	20.96%	31.06%	14.85%
	Others	9.56%	6.91%	8.73%	5.80%
	**2000-2009**				
Self-supported	Self-employed (≥10 employees)	0.13%	0.13%	0.08%	0.14%
	Self-employed (5-9 employees)	0.28%	0.35%	0.24%	0.39%
	Self-employed (1-4 employees)	2.16%	2.64%	1.79%	2.92%
	Self-employed (0 employees)	5.17%	5.75%	6.22%	5.60%
	Assisting spouses	0.48%	0.58%	0.20%	0.70%
	Employee with management work	3.18%	2.29%	2.39%	2.26%
	Employee in work that requires skills at the highest level	14.01%	9.29%	13.05%	8.07%
	Employee in work that requires skills at medium level	19.00%	15.53%	17.27%	14.97%
	Employee in work that requires skills at the basic level	36.84%	41.91%	35.40%	44.02%
	Other employees	7.88%	9.95%	8.75%	10.34%
	Employee, position statement not stated	10.86%	11.57%	14.60%	10.58%

Public supported	Unemployed at least half of the year	12.90%	10.84%	11.53%	10.41%
	Sickness benefits, leave, etc.	7.15%	5.36%	6.14%	4.88%
	Students	4.80%	2.47%	3.72%	1.69%
	Non-old age pensioners	35.61%	42.91%	39.87%	44.79%
	Old age pensioners	0.00%	0.00%	0.00%	0.00%
	Early retirement scheme	17.06%	17.13%	9.89%	21.63%
	Recipients of cash benefits	13.61%	14.34%	21.87%	9.66%
	Others	8.87%	6.95%	6.97%	6.93%
	**1992-1999**				
Self-supported	Self-employed (≥10 employees)	0.13%	0.13%	0.08%	0.14%
	Self-employed (5-9 employees)	0.28%	0.35%	0.24%	0.39%
	Self-employed (1-4 employees)	2.16%	2.64%	1.79%	2.92%
	Self-employed (0 employees)	5.17%	5.75%	6.22%	5.60%
	Assisting spouses	0.48%	0.58%	0.20%	0.70%
	Employee with management work	3.18%	2.29%	2.39%	2.26%
	Employee in work that requires skills at the highest level	14.01%	9.29%	13.05%	8.07%
	Employee in work that requires skills at medium level	19.00%	15.53%	17.27%	14.97%
	Employee in work that requires skills at the basic level	36.84%	41.91%	35.40%	44.02%
	Other employees	7.88%	9.95%	8.75%	10.34%
	Employee, position statement not stated	10.86%	11.57%	14.60%	10.58%
Public supported	Unemployed at least half of the year	12.90%	10.84%	11.53%	10.41%
	Sickness benefits, leave, etc.	7.15%	5.36%	6.14%	4.88%
	Students	4.80%	2.47%	3.72%	1.69%
	Non-old age pensioners	35.61%	42.91%	39.87%	44.79%
	Old age pensioners	0.00%	0.00%	0.00%	0.00%
	Early retirement scheme	17.06%	17.13%	9.89%	21.63%
	Recipients of cash benefits	13.61%	14.34%	21.87%	9.66%
	Others	8.87%	6.95%	6.97%	6.93%

Appendix Table 2. Person-years, number of deaths and mortality rate ratio for people aged 30-64 years in Lolland-Falster by residency group, employment status, sex, and calendar year period. Mortality rate ratios were adjusted for 5-year age groups and sex. Self-supported and public supported, respectively, in the rest of Denmark is the reference population.

	**Self-supported**			**Public support**		
	Person-years	Deaths	MRR	Person-years	Deaths	MRR
**2010-2018**						
Men						
Denmark	8646953	15626	1	2069044	28895	1
Lolland-Falster	152071	431	1.41 (1.28-1.56)	64748	1168	1.29 (1.19-1.39)
In-migrants	38164	91	1.76 (1.43-2.17)	25747	440	1.43 (1.24-1.64)
Long-term residents	113907	340	1.34 (1.21-1.49)	39001	728	1.24 (1.13-1.35)
Women						
Denmark	8126114	9415	1	2568593	18162	1
Lolland-Falster	137546	249	1.38 (1.22-1.57)	69904	671	1.27 (1.20-1.35)
In-migrants	30943	35	1.33 (0.96-1.86)	24987	202	1.51 (1.38-1.66)
Long-term residents	106603	214	1.39 (1.21-1.59)	44918	469	1.16 (1.07-1.24)
Total						
Denmark	16773066	25041	1	4637636	47057	1
Lolland-Falster	289617	680	1.40 (1.30-1.51)	134652	1839	1.28 (1.22-1.34)
In-migrants	69107	126	1.62 (1.36-1.93)	50733	642	1.49 (1.38-1.61)
Long-term residents	220510	554	1.36 (1.25-1.48)	83919	1197	1.19 (1.12-1.26)
**2000-2009**						
Men						
Denmark	10053788	23997	1	2328834	38053	1
Lolland-Falster	204578	679	1.31 (1.21-1.41)	75642	1567	1.23 (1.17-1.29)
In-migrants	52121	163	1.76 (1.51-2.05)	31421	638	1.38 (1.28-1.49)
Long-term residents	152458	516	1.21 (1.11-1.32)	44220	929	1.14 (1.07-1.22)
Women						
Denmark	8990465	12973	1	3185614	25792	1
Lolland-Falster	176145	327	1.19 (1.07-1.33)	90747	909	1.19 (1.12-1.28)
In-migrants	41097	65	1.47 (1.16-1.88)	32412	314	1.41 (1.26-1.57)
Long-term residents	135048	262	1.14 (1.01-1.29)	58335	595	1.11 (1.02-1.20)
Total						
Denmark	19044254	36970	1	5514448	63845	1

Lolland-Falster	380723	1006	1.27 (1.19-1.35)	166389	2476	1.22 (1.17-1.27)
In-migrants	93218	228	1.67 (1.46-1.90)	63833	952	1.39 (1.30-1.48)
Long-term residents	287505	778	1.18 (1.10-1.27)	102556	1524	1.13 (1.07-1.19)
**1992-1999**						
Men						
Denmark	7351405	20888	1	1929695	31394	1
Lolland-Falster	159552	509	1.09 (0.99-1.19)	62537	1080	1.03 (0.97-1.09)
In-migrants	33619	92	1.30 (1.06-1.59)	24476	403	1.12 (1.01-1.23)
Long-term residents	125932	417	1.05 (0.95-1.15)	38061	677	0.98 (0.91-1.06)
Women						
Denmark	6309543	10657	1	2820938	23463	1
Lolland-Falster	132161	259	1.12 (0.99-1.27)	82652	727	1.01 (0.94-1.09)
In-migrants	25323	44	1.44 (1.07-1.93)	24906	208	1.18 (1.03-1.35)
Long-term residents	106839	215	1.08 (0.94-1.23)	57745	519	0.96 (0.88-1.05)
Total						
Denmark	13660948	31545	1	4750633	54857	1
Lolland-Falster	291713	768	1.10 (1.02-1.18)	145189	1807	1.02 (0.98-1.07)
In-migrants	58942	136	1.34 (1.13-1.58)	49382	611	1.14 (1.05-1.23)
Long-term residents	232771	632	1.06 (0.98-1.14)	95807	1196	0.97 (0.92-1.03)

Appendix Table 3. Person-years, number of deaths and mortality rate ratio for people aged 30-64 years in Lolland-Falster by residency group, employment status, sex, and calendar year period. Mortality rate ratios were adjusted for 5-year age groups and sex. Self-supported in rest of Denmark is the reference population. Residency groups based on 20 years moving history before time period.

	**Self-supported**			**Public support**			**Total**		
	Person-years	Deaths	MRR	Person-years	Deaths	MRR	Person-years	Deaths	MRR
**2010-2018 (residency group 20 years prior)**									
Men									
Denmark	7642128	13535	1	1828408	25189	6.27 (6.14-6.41)	9470536	38724	2.16 (2.12-2.20)
Lolland-Falster	132108	373	1.46 (1.31-1.61)	55503	985	7.97 (7.47-8.50)	187611	1358	3.44 (3.25-3.63)
In-migrants	48737	113	1.57 (1.30-1.89)	29679	529	9.72 (8.92-10.61)	78416	642	5.12 (4.73-5.54)
Long-term residents	83372	260	1.41 (1.25-1.60)	25824	456	6.58 (6.00-7.23)	109196	716	2.65 (2.46-2.86)
Women									
Denmark	7179136	8197	1	2279666	15790	4.80 (4.67-4.94)	9458802	23987	2.02 (1.97-2.07)
Lolland-Falster	118906	222	1.46 (1.28-1.67)	59847	572	6.25 (5.74-6.81)	178753	794	3.08 (2.87-3.32)
In-migrants	42823	60	1.51 (1.17-1.95)	29654	255	7.16 (6.32-8.11)	72477	315	4.15 (3.71-4.64)
Long-term residents	76083	162	1.44 (1.23-1.68)	30193	317	5.68 (5.07-6.35)	106276	479	2.64 (2.40-2.89)
Total									
Denmark	14821264	21732	1	4108075	40979	5.68 (5.59-5.78)	18929338	62711	2.10 (2.07-2.14)
Lolland-Falster	251015	595	1.46 (1.34-1.58)	115349	1557	7.30 (6.94-7.69)	366364	2152	3.30 (3.16-3.45)
In-migrants	91559	173	1.54 (1.33-1.79)	59333	784	8.74 (8.14-9.38)	150893	957	4.75 (4.46-5.07)
Long-term residents	159455	422	1.42 (1.29-1.57)	56016	773	6.26 (5.83-6.73)	215471	1195	2.65 (2.50-2.81)
**2000-2009 (residency group 20 years prior)**									
Men									
Denmark	10053963	23999	1	2328918	38058	5.37 (5.28-5.46)	12382881	62057	1.92 (1.89-1.95)
Lolland-Falster	203610	675	1.31 (1.21-1.41)	74693	1547	6.57 (6.24-6.92)	278303	2222	2.80 (2.68-2.93)
In-migrants	73124	221	1.49 (1.31-1.70)	39670	816	7.47 (6.96-8.01)	112794	1037	3.95 (3.71-4.20)
Long-term residents	130486	454	1.24 (1.13-1.36)	35023	731	5.80 (5.39-6.24)	165509	1185	2.24 (2.11-2.37)
Women									
Denmark	8990531	12973	1	3185639	25791	4.23 (4.14-4.33)	12176169	38764	1.89 (1.86-1.93)
Lolland-Falster	175135	326	1.20 (1.07-1.34)	89715	896	5.04 (4.71-5.39)	264850	1222	2.50 (2.35-2.65)
In-migrants	62085	104	1.39 (1.15-1.68)	41357	407	6.02 (5.45-6.64)	103442	511	3.43 (3.14-3.74)
Long-term residents	113050	222	1.13 (0.99-1.29)	48358	489	4.43 (4.05-4.86)	161408	711	2.09 (1.94-2.25)
Total									
Denmark	19044493	36972	1	5514557	63849	4.93 (4.86-4.99)	24559050	100821	1.91 (1.89-1.93)
Lolland-Falster	378745	1001	1.27 (1.19-1.35)	164408	2443	5.99 (5.75-6.24)	543154	3444	2.69 (2.60-2.78)

In-migrants	135209	325	1.45 (1.30-1.62)	81027	1223	6.94 (6.56-7.35)	216236	1548	3.76 (3.57-3.95)
Long-term residents	243536	676	1.20 (1.11-1.29)	83381	1220	5.26 (4.97-5.57)	326917	1896	2.18 (2.08-2.29)
**1992-1999 (residency group 20 years prior)**									
Men									
Denmark	7351462	20889	1	1929823	31396	3.38 (3.30-3.46)	9281285	52285	1.77 (1.74-1.80)
Lolland-Falster	158981	509	1.13 (1.00-1.28)	62275	1074	3.41 (3.16-3.68)	221256	1583	2.09 (1.99-2.20)
In-migrants	56520	144	1.17 (0.94-1.47)	31146	529	3.96 (3.52-4.46)	87666	673	2.71 (2.51-2.93)
Long-term residents	102462	365	1.11 (0.96-1.29)	31129	545	3.13 (2.85-3.45)	133590	910	1.79 (1.67-1.91)
Women									
Denmark	6309596	10657	1	2820973	23466	4.30 (4.22-4.38)	9130569	34123	1.80 (1.76-1.84)
Lolland-Falster	131512	258	1.10 (1.00-1.20)	82151	721	4.41 (4.14-4.69)	213663	979	2.02 (1.89-2.16)
In-migrants	46867	76	1.04 (0.88-1.22)	33683	279	5.05 (4.63-5.50)	80550	355	2.52 (2.27-2.80)
Long-term residents	84645	182	1.12 (1.01-1.24)	48468	442	3.92 (3.60-4.27)	133113	624	1.81 (1.67-1.97)
Total									
Denmark	13661058	31546	1	4750797	54862	3.95 (3.89-4.00)	18411854	86408	1.78 (1.75-1.80)
Lolland-Falster	290493	767	1.11 (1.03-1.19)	144427	1795	4.02 (3.84-4.22)	434919	2562	2.06 (1.98-2.15)
In-migrants	103387	220	1.08 (0.94-1.23)	64829	808	4.65 (4.34-4.99)	168216	1028	2.64 (2.48-2.81)
Long-term residents	187106	547	1.12 (1.03-1.22)	79597	987	3.62 (3.40-3.86)	266703	1534	1.80 (1.71-1.89)

Appendix Table 4. Person-years, number of deaths and mortality rate ratio for people aged 30-64 years in Lolland-Falster by residency group, employment status (annual status three years back in time), sex, and calendar year period. Mortality rate ratios were adjusted for 5-year age groups and sex. Self-supported in rest of Denmark is the reference population.

	**Self-supported (three years prior)**			**Public support (three years prior)**			**Total**		
	Person-years	Deaths	MRR	Person-years	Deaths	MRR	Person-years	Deaths	MRR
**2010-2018**									
Men									
Denmark	8906183	21175	1	1795766	23357	5.14 (5.04-5.23)	10701949	44532	1.77 (1.74-1.80)
Lolland-Falster	162174	612	1.43 (1.32-1.55)	54619	988	6.71 (6.29-7.15)	216792	1600	2.09 (1.99-2.20)
In-migrants	39998	141	1.92 (1.63-2.27)	23891	389	7.94 (7.19-8.78)	63889	530	3.03 (2.77-3.32)
Long-term residents	122176	471	1.33 (1.21-1.45)	30727	599	6.09 (5.62-6.61)	152903	1070	1.83 (1.72-1.95)
Women									
Denmark	8417715	13152	1	2265298	14412	3.84 (3.75-3.93)	10683013	27564	1.80 (1.76-1.84)
Lolland-Falster	148348	356	1.35 (1.22-1.50)	59061	563	5.14 (4.72-5.59)	207408	919	2.02 (1.89-2.16)
In-migrants	32695	61	1.60 (1.24-2.05)	23199	176	5.53 (4.76-6.41)	55894	237	2.82 (2.49-3.19)
Long-term residents	115653	295	1.31 (1.17-1.47)	35861	387	4.98 (4.50-5.51)	151514	682	1.84 (1.71-1.99)
Total									
Denmark	17323898	34327	1	4061064	37769	4.60 (4.53-4.67)	21384962	72096	1.78 (1.76-1.80)
Lolland-Falster	310521	968	1.40 (1.31-1.49)	113679	1551	6.08 (5.78-6.40)	424200	2519	2.06 (1.98-2.15)
In-migrants	72693	202	1.80 (1.57-2.07)	47090	565	7.00 (6.44-7.61)	119783	767	2.96 (2.75-3.18)
Long-term residents	237828	766	1.32 (1.23-1.42)	66589	986	5.66 (5.31-6.03)	304417	1752	1.84 (1.75-1.93)
**2000-2009**									
Men									
Denmark	10221483	31312	1	2146874	30720	4.24 (4.17-4.31)	12368356	62032	1.62 (1.59-1.65)
Lolland-Falster	211824	951	1.37 (1.28-1.46)	68303	1294	5.13 (4.86-5.43)	280127	2245	2.47 (2.35-2.60)
In-migrants	52741	265	2.08 (1.85-2.35)	30716	537	5.63 (5.17-6.14)	83457	802	3.75 (3.46-4.06)
Long-term residents	159082	686	1.21 (1.12-1.30)	37587	757	4.83 (4.49-5.19)	196670	1443	2.04 (1.92-2.18)
Women									
Denmark	9063457	17423	1	3100326	21315	3.12 (3.06-3.18)	12163783	38738	1.57 (1.54-1.61)
Lolland-Falster	180497	448	1.20 (1.09-1.31)	86323	789	3.82 (3.55-4.10)	266820	1237	2.14 (2.00-2.29)
In-migrants	40601	93	1.53 (1.25-1.88)	32843	287	4.64 (4.13-5.22)	73444	380	3.22 (2.87-3.61)
Long-term residents	139896	355	1.13 (1.02-1.26)	53481	502	3.46 (3.17-3.79)	193376	857	1.82 (1.67-1.98)

Total									
Denmark	19284939	48735	1	5247199	52035	3.78 (3.73-3.83)	24532139	100770	1.60 (1.58-1.62)
Lolland-Falster	392320	1399	1.31 (1.24-1.38)	154626	2083	4.61 (4.42-4.82)	546946	3482	2.35 (2.25-2.44)
In-migrants	93342	358	1.90 (1.71-2.10)	63558	824	5.27 (4.92-5.65)	156901	1182	3.56 (3.33-3.80)
Long-term residents	298978	1041	1.18 (1.11-1.25)	91068	1259	4.27 (4.03-4.51)	390046	2300	1.96 (1.86-2.06)

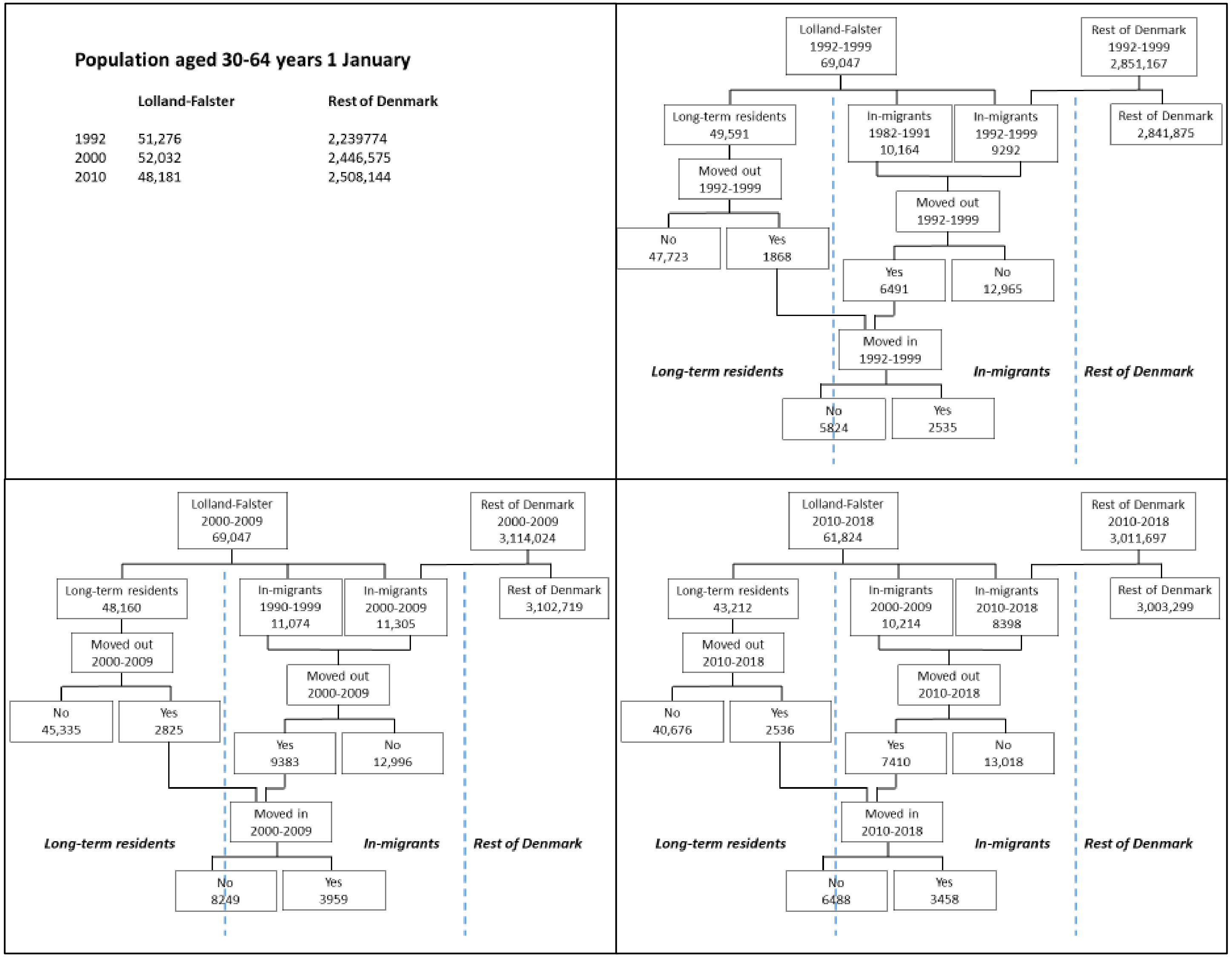

Appendix Figure 1. Number of people aged 30-64 years during a time-period by residency groups for 1992-1999, 2000-2009, and 2010-2018. During a time-period a person could move downwards in the flow diagram if they moved between Lolland-

Falster and the rest of Denmark. People entered the analysis as they reached age 30 years and exited the analysis as they reached age 65 years.
